# Health beliefs, lifestyle, and cognitive aging among Chinese community residents: A structural equation model analysis

**DOI:** 10.3389/fpubh.2022.1028679

**Published:** 2022-12-02

**Authors:** Jinying Zhang, Xiao Liu, De Gong, Yan Peng, Hua Li, Yanni Yang

**Affiliations:** School of Nursing, Third Military Medical University/Army Medical University, Chongqing, China

**Keywords:** Health Belief Model, lifestyle, cognitive aging, structural equation modeling, middle-aged and older adults

## Abstract

**Background:**

Lifestyle factors may could help maintain cognitive function and reduce the risk of dementia. The application of the Health Belief Model (HBM) has been verified by incorporating lifestyle changes for dementia risk reduction; however, the influence of health beliefs on cognitive aging through lifestyle remains unknown. To facilitate research-based interventions to promote successful cognitive aging, we explored the relationship between health beliefs, lifestyle, and cognitive aging based on the HBM using path analysis.

**Methods:**

This cross-sectional study recruited middle-aged and older community residents from a community health service center in Chongqing, China, through convenience sampling. Motivation to Change Lifestyle and Health Behaviors for Dementia Risk Reduction (MCLHB-DRR), Lifestyle for Dementia Risk Reduction (LDRR), and the Montreal Cognitive Assessment (MoCA) were employed to measure participants' beliefs, lifestyle, and cognitive function, respectively. The associations between the beliefs, lifestyle, and cognitive function were analyzed, and a structural equation model was constructed.

**Results:**

A total of 202 participants completed the questionnaires, of whom only 17 (8.4%) were classified as having successful cognitive aging. The model demonstrated the data to have an acceptable fit and elucidated 39.3 and 18.2% of the variance in lifestyle and the grade of cognitive aging, respectively. Positive and negative beliefs had opposite effects on the grade of cognitive aging through lifestyle. Cues to action had opposite effects on the grades of cognitive aging through positive and negative beliefs; however, the total effects canceled each other out.

**Conclusions:**

Positive beliefs have a positive effect on lifestyle, thereby promoting successful cognitive aging, whereas negative beliefs have a negative effect on lifestyle, thereby hindering successful cognitive aging. Health education and media publicity, as specific aspects of cues to action, can have a meaningful impact on healthy behavior and successful cognitive aging by promoting positive beliefs and controlling negative beliefs. The model suggests the strengthening and weakening of the positive and negative beliefs, respectively, of middle-aged and older community residents in the formulation of relevant public health strategies in the future, thereby enabling them to adapt to a healthy lifestyle promoting successful cognitive aging.

## Introduction

Cognitive aging describes the transformation of cognitive function with aging, which is characterized by a decline in attention, information processing speed, executive function, and episodic memory (1). Usually, cognitive decline is slow and acceptable, and does not seriously affect older adults' daily life. Unusual cognitive aging is associated with a variety of neurological diseases, especially dementia, which has been a major cause of disability in older adults and exerts a considerable burden on society and the economy (2). With increase in global aging, dementia has increased rapidly in recent years. An estimated 50 million people worldwide were living with dementia in 2018, and this number is expected to increase to 152 million by 2050 (3). Although dementia is characterized by symptoms of cognitive dysfunction, it is not an inevitable consequence of cognitive aging. Multiple factors result in cognitive dysfunction, which is difficult to reverse; however, its preventability is being explored and supported by an increasing amount of evidence.

It's not just cognitive dysfunction that needs to be improved, and usual cognitive aging also wouldn't be the ultimate goal of cognitive interventions in older adults. Despite not reaching the stage of cognitive dysfunction, significant differences exist among individuals in terms of cognitive aging. Based on the heterogeneity of cognitive aging (4), Rowe and Kahn (5) proposed the concept of successful cognitive aging, which differs from the concept of usual cognitive aging and highlights a higher level of cognitive function preservation and improvement. In the study by Hartley et al. (6), cognitive aging was classified into three grades: unsuccessful, usual, and successful. Exploring the common characteristics of successful cognitive aging could be helpful in providing targets for cognitive intervention and facilitating the individual's transition to successful cognitive aging.

Previous studies have found that usual cognitive aging may be influenced by certain physiological, psychological, behavioral, and social factors; modifying these factors could aid in the prevention or delay of cognitive decline and further prevent dementia (7). Approximately 40% of dementia is attributable to a combination of the following 12 risk factors: less education, hypertension, hearing impairment, smoking, obesity, depression, physical inactivity, diabetes, low social contact, alcohol consumption, traumatic brain injury, and air pollution (8). It is widely recommended to maintain a healthy lifestyle (9), which can help maintain a higher level of cognitive and physical function and reduce neuropathological damage in neurodegenerative diseases associated with cognitive aging (10). Therefore, we propose in hypothesis 1 that adherence to a cognitive-related lifestyle promotes cognitive aging. However, the lifestyle of most people still falls short of the recommended guidelines owing to various barriers between theoretical guidance and practicality (11). Therefore, exploring the internal factors of people's adoption of cognitive-related healthy lifestyles and providing evidence for developing specific intervention strategies are necessary for successful cognitive aging.

The Health Belief Model (HBM) could be helpful in understanding the beliefs and motivations for adopting a cognitive-related healthy lifestyle. The HBM, which is one of the earliest and most widely used theories in the field of health behavior, is capable of exploring internal factors and elucidating the internal decision-making process of the individual's health behavior. HBM is most commonly used for prevention-related and asymptomatic health problems, such as early cancer detection and hypertension screening, in which beliefs are just as or more important than obvious symptoms (12). Six dimensions were described in the HBM, including perceived susceptibility, perceived severity, perceived benefits, perceived barriers, cues to action, and self-efficacy. Compared with five other behavioral change models (e.g., Health Locus of Control, Theory of Reasoned Action/Theory of Planned Behavior, Self-efficacy Theory, Stage of Change/Transtheoretical Model of Change, and Common Sense Model of Self-regulation), the HBM was considered the best model to elucidate the mechanism of risk reduction of dementia (13). Thus, exploring the related mechanism from the perspective of the HBM is important when establishing a healthy lifestyle for successful cognitive aging.

Previous studies have focused more on the status and influencing factors of dementia prevention beliefs and their correlation with willingness to engage in health-related behaviors (14–17). Akyol et al. (16) conducted a survey on the dementia prevention beliefs of 284 Turkish people over the age of 40 years; the results demonstrated that age, years of education, family history of dementia, subjective memory complaints, and willingness to understand their own risks were influencing factors of dementia prevention beliefs. Moreover, a study by Seifan et al. (15) demonstrated that individuals' beliefs in dementia prevention would affect their willingness to engage in health-related behaviors, such as actively seeking help from professional doctors, actively identifying personal risk of dementia, and actively learning knowledge of dementia prevention. However, the effect of the individuals' beliefs on cognitive-related behaviors and the characteristics of beliefs deserve attention in dementia prevention. In a recent study, Li et al. (18) found that different health beliefs had either positive or negative effects on health-promoting lifestyle. However, the scale used in Li et al. (18) study was the Chinese version of the Health Promoting Lifestyle Profile-II (HPLP-II), which was lack of pertinence to assess the lifestyles that reduce the risk of dementia. In addition, the belief-lifestyle exploration in this study did not extend to the final cognitive outcome. The contribution of beliefs in dementia prevention on cognitive aging by influencing cognitive-related healthy lifestyles should be explored more deeply.

In this study, our classification of health beliefs referred to the findings revealed by Li et al. (18). Furthermore, according to the HBM (19), as an external cause, cues to action can directly affect health behaviors or indirectly affect health behaviors by acting on other belief dimensions. Based on the study of Li et al. (18), in combination with the theory of HBM, the health belief was re-divided into three dimensions in this study: positive beliefs, negative beliefs, and cues to action. Therefore, we propose in hypothesis 2 that positive beliefs have a promoting effect on lifestyle, negative beliefs have a hindering effect on lifestyle, and cues to action can directly affect lifestyle, and can also indirectly affect lifestyle through positive and negative beliefs. The Reduced Risk of Dementia Lifestyle Measure (LDRR) (20), which measures cognitive-related healthy lifestyles, was designed in our previous studies based on the HPLP-II and the latest evidence on dementia prevention, and has been verified to have good reliability and validity. The LDRR focusing on multiple healthy lifestyles that reduce the risk of dementia, and is suitable for this study to explore the relationship between beliefs, lifestyles and cognitive aging. We propose in hypothesis 3 that health beliefs affect cognitive aging by influencing cognitive-related healthy lifestyle.

**Figure 1:**
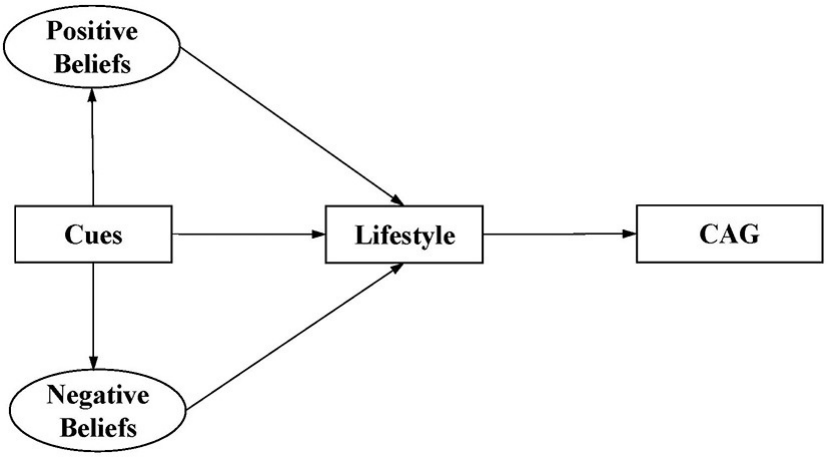
Constructed model for hypothesis testing.

Based on the HBM, this study aimed to describe the status of dementia prevention beliefs of the individuals and establish a structural model of beliefs, lifestyle, and cognitive aging. This study aimed to elucidate whether dementia-prevention beliefs have a direct impact on cognitive-related healthy lifestyles; successful cognitive aging is related to a healthy cognitive-related lifestyle; and dementia-prevention beliefs have an indirect effect on cognitive aging through lifestyle. Figure 1 shows our constructed model for the hypothesis testing.

## Materials and methods

### Study design

This cross-sectional study employed a structural equation model.

### Study setting

A questionnaire-based survey was conducted from March to October 2021 in a community health service center in Chongqing, China. The community health service center has jurisdiction over five communities with a total area of 2.9 km^2^ and permanent residents over 56,000.

### Participants

Through convenience sampling, middle-aged and older adults who participated in free physical examinations at the community health service center were recruited. The inclusion criteria were as follows: (1) age ≥45 years, (2) attended free physical examination and established health records at the community health service center, (3) gave informed consent, and (4) had normal communication and comprehension skills, and able to read and fill in the required questionnaire independently (or through the researcher). The exclusion criteria were as follows: (1) had a clear diagnosis of various types of dementia, (2) inability to complete the investigation owing to serious physical illness, and (3) had severe visual or hearing impairment and inability to communicate normally.

### Data collection

All the data collectors accepted uniform training before the study to ensure familiarity with all the questionnaires and the survey process and avoid bias during data collection. The participants completed the questionnaires with the assistance of the data collectors only after they received a clear statement of the study's objectives and meaning and provided written informed consent. Incomplete questionnaires triggered a second interview. Participants who could not be re-contacted were excluded from the study. Since the sample size required to construct the structural equation model is at least 100, preferably 200 or more (21), a total of 221 adults were invited to participate in this study, and 202 (91.4%) completed all the questionnaires. This study was approved by the Medical Ethics Committee of Army Medical University (2021 No.18-02).

### Measures

#### Demographic characteristic questionnaire

The participants' general characteristics, including gender, age, education level, marital status, living conditions, personal monthly income, family history, and contact history of dementia, were self-reported by the participants based on the questionnaire.

#### Motivation to change lifestyle and health behaviors for dementia risk reduction

The MCLHB-DRR is a 27-item scale developed by Kim et al. (13) in 2014 to measure beliefs about lifestyle and behavioral changes for dementia risk reduction. Seven subscales were included: perceived susceptibility (four items), perceived severity (five items), perceived benefits (four items), perceived barriers (four items), cues to action (four items), general health motivation (four items), and self-efficacy (two items). All the items were rated on a 5-point Likert scale ranging from 1 (strongly disagree) to 5 (strongly agree). A higher score indicates a higher level of motivation to change their lifestyle. The scale was cross-culturally adapted in our previous study (22), and the Chinese version has been validated, which showed that the Kaiser–Meyer–Olkin value of the scale was 0.74, and the Cronbach's alpha for the scale was 0.76.

#### Lifestyle for dementia risk reduction

The LDRR (20) was designed in our previous studies to assess an individual's lifestyle related to dementia risk reduction. This is a 32-item scale with eight dimensions: health responsibility (four items), brain-benefiting exercise (five items), mental leisure activity (two items), brain-benefiting diet (five items), tobacco control behavior (two items), interpersonal relationship (five items), stress management (four items), and spiritual growth (five items). Items were rated on a 4-point Likert scale from 1 (never) to 4 (always), while one was scored in reverse. The total score ranges from 32 to 128, with a higher score indicating a healthier lifestyle to reduce the risk of dementia. This finding has been validated in the Chinese population. Exploratory factor analysis showed that the cumulative variance contribution rate was 60.189% and the factor loadings ranged from 0.403 to 0.866. The fitness indices of the confirmatory factor analysis reached acceptable levels. Moreover, Cronbach's alpha of this scale was 0.855, and its test-retest reliability was 0.864.

####  Montreal cognitive assessment

The MoCA, developed by Nasreddine et al. (23) in 2004, measures eight cognitive domains: orientation, language, working memory, concentration, short-term memory, attention, executive function, and visuospatial ability. The MoCA is a widely used screening tool for general cognitive function, with a total score of 30; a higher score indicates a higher level of cognitive function. The Beijing version (24) of the MoCA used in this study has been modified from a cultural and linguistic perspective. The sensitivity of the Beijing version of the MoCA was 83.8, 80.5, and 96.9% for all cognitive impairments, mild cognitive impairments, and dementia, respectively; the specificity for identifying cognitively normal was 82.5% (25).

Based on a study by Hartley et al. (6), cognitive aging was divided into three grades in different age groups. Age was divided into several groups with the 10-year-old range in this study, including 45–59 (since the range of 45–49 years old is less than 10 years old, this age group was combined with 50–59 years old), 60–69, 70–79, and 80–89 years. Moreover, the standard score of MoCA was used, which was calculated using the original MoCA score and the mean and standard deviation (SD) scores, as follows: standard score = (MoCA-mean)/SD. Participants whose standard scores were more than 1 standard deviation above the mean in their age group would be classified as “successful cognitive aging” (Score>Mean+1 × SD); those with more than 1 standard deviation below the mean in their age group were “unsuccessful cognitive aging” (Score < Mean-1 × SD); and those in between were “usual cognitive aging” (Mean-1 × SD ≤ Score ≤ Mean+1 × SD).

### Statistical analysis

SPSS software (version 24.0) was used for statistical analysis. Statistical significance was set at *p* ≤ 0.05. Frequency and percentage were used to describe participant demographics. The mean and SD or median and interquartile range (IQR) was used to describe the MCLHB-DRR, LDRR, and MoCA scores. The Mann–Whitney U test and Kruskal–Wallis *H-*test were used to test the differences in cognitive aging grades for each demographic characteristic. After that, Bonferroni correction method was used for pairwise comparison test for variables with three categories and above. Spearman correlation coefficient was used to test the associations between various health beliefs and lifestyles. Version 8.3 of Mplus software was used for path analysis and construction of the structural equation model as well as for the confirmatory factor analysis (CFA) of the measurement model. Before running CFA, we used Henze-Zirkler (HZ) test to test the multivariate normality of the variables in Stata software (version 17.0). Maximum likelihood (ML) method was used for CFA if it is consistent with multivariate normal distribution, otherwise, maximum likelihood parameter estimates with standard errors and a mean-adjusted chi-square test statistic (MLM) method was used for CFA. Considering the classified outcome variables used in this study, weighted least squares with mean and variance adjusted (WLSMV) estimation, instead of maximum likelihood (ML) estimation, was used to estimate the model. Considering the classified outcome variables, WLSMV is considered a suitable estimation and better than ML (26, 27). The comparative fit index (CFI), Tucker–Lewis index (TLI), and root mean square error of approximation (RMSEA) were used to evaluate the goodness of fit of the model. Acceptable model fitting was determined by RMSEA < 0.08 and CFI and TLI values > 0.90 (28).

## Results

### Demographic characteristics and cognitive aging grades

A total of 202 participants provided their complete data. The mean age of the participants was 67.10 ± 7.62 years old. Most participants were female (132, 65.3%), married (157, 77.7%), living with someone (180, 89.1%), had a primary education or below (60, 29.7%), and had a monthly income of 1000 RMB (22, 10.9%). Furthermore, only 21 (10.4%) participants had a family history of dementia, while nearly half of the participants (100, 49.5%) had prior contact with patients with dementia.

Considering the cognitive aging grades, there were 17 (8.4%), 155 (76.7%), and 30 (14.9%) participants in the “successful cognitive aging,” “usual cognitive aging,” and “unsuccessful cognitive aging” groups, respectively. Statistically significant differences in the cognitive aging grades were observed among participants with different educational levels (*Hc* = 39.655, *p* < 0.001) and personal monthly incomes (*Hc* = 14.756, *p* = 0.001). Further pairwise comparison test showed that the cognitive aging grade was lower in those with primary school education or below (*p* < 0.05), and personal monthly incomes < 1000 RMB. The detailed data are presented in Table 1.

**Table 1:** Sociodemographic and cognitive aging characteristics [*n* (%)].

**Characteristics**	**All (*n =* 202)**	**Successful group (*n =* 17)**	**Normal group (*n =* 155)**	**Impaired group (*n =* 30)**	**Mean of rank**	***Z*/*Hc***	***P***
Gender						−1.176^a^	0.240
Male	70 (34.7)	3 (1.5)	63 (31.2)	4 (2.0)	106.40		
Female	132 (65.3)	14 (6.9)	92 (45.5)	26 (12.9)	98.90		
Age group (years)						0.464^b^	0.927
45~59	36 (17.8)	5 (2.5)	24 (11.9)	7 (3.5)	101.96		
60~69	95 (47.0)	11 (5.4)	69 (34.2)	15 (7.4)	103.35		
70~79	60 (29.7)	1 (0.5)	52 (25.7)	7 (3.5)	98.64		
80~89	11 (5.5)	0 (0.0)	10 (5.0)	1 (0.5)	99.59		
Education level						39.655^b^	< 0.001
Primary or below	60 (29.7)	0 (0.0)	37 (18.3)	23 (11.4)	72.54		
Middle school	74 (36.6)	6 (3.0)	64 (31.7)	4 (2.0)	109.97^c^		
Senior high school or above	68 (33.7)	11 (5.4)	54 (26.7)	3 (1.5)	117.83^c^		
Marital status						−1.856^a^	0.063
Married	157 (77.7)	13 (6.4)	126 (62.4)	18 (8.9)	104.52		
Single/divorced/widowed	45 (22.3)	4 (2.0)	29 (14.4)	12 (5.9)	90.98		
Living conditions						−0.647^a^	0.518
Living alone	22 (10.9)	3 (1.5)	16 (7.9)	3 (1.5)	107.11		
Other	180 (89.1)	14 (6.9)	139 (68.8)	27 (13.4)	100.81		
Individual monthly income (RMB)*						14.756^b^	0.001
< 1000	22 (10.9)	1 (0.5)	11 (5.4)	10 (5.0)	69.86		
1000~3000	72 (35.6)	4 (2.0)	52 (25.7)	16 (7.9)	92.22^c^		
3000~5000	86 (42.6)	10 (5.0)	72 (35.6)	4 (2.0)	113.70^cd^		
>5000	22 (10.9)	2 (1.0)	20 (9.9)	0 (0.0)	115.82^cd^		
Family history of dementia						−0.788^a^	0.430
No	181 (89.6)	16 (7.9)	139 (68.8)	26 (12.9)	102.31		
Yes	21 (10.4)	1 (0.5)	16 (7.9)	4 (2.0)	94.48		
Contact with dementia patients						−0.140^a^	0.888
No	102 (50.5)	9 (4.5)	77 (38.1)	16 (7.9)	101.08		
Yes	100 (49.5)	8 (4.0)	78 (38.6)	14 (6.9)	101.93		

*One RMB was about 0.15 USD. ^a^Mann-Whitney U-test, ^b^Kruskal-Wallis H-test, ^c^Represents *p* < 0.05 compared with the first layer, ^d^Represents *p* < 0.05 compared with the second layer. Bonferroni correction method was used for pairwise comparison test.

### Bivariate correlations between health beliefs and lifestyle

The mean lifestyle score in this study was 89.71 (SD = 11.61). The mean scores for each health belief subscale are shown in Table 2. In this study, perceived susceptibility and perceived barriers negatively correlated with lifestyle, while perceived benefits, general health motivation, and self-efficacy positively correlated with lifestyle (Table 3). Perceived severity was positively correlated with perceived susceptibility (*R* = 0.265, *p* < 0.001) and perceived barriers (*R* = 0.229, *p* < 0.01), which are negative beliefs. Perceived severity is also a perceived threat, similar to perceived susceptibility. Thus, although the correlation coefficient between perceived severity and lifestyle was not statistically significant, it would be better to consider perceived severity as a negative belief, together with perceived susceptibility and perceived barriers in this study.

**Table 2:** Descriptive statistics of health beliefs and lifestyle (*N* = 202).

**Variable**	**Range**	**Min**	**Max**	**Mean ±SD/Median (IQR)**
Health beliefs	27~135	67.00	129.00	102.70 ± 12.15
Perceived susceptibility	4~20	4.00	20.00	10.00 (6.00–12.00)
Perceived severity	5~25	5.00	25.00	19.00 (14.00–22.25)
Perceived benefits	4~20	8.00	20.00	20.00 (18.00-20.00)
Perceived barriers	4~20	4.00	20.00	7.00 (4.00–12.00)
General health motivation	4~20	6.00	20.00	20.00 (18.00–20.00)
Self-efficiency	2~10	2.00	10.00	10.00 (8.00–10.00)
Cues to action	4~20	4.00	20.00	10.00 (14.00–16.00)
Lifestyle	32~128	62.00	117.00	89.71 ± 11.61

**Table 3:** Correlations between variables of health beliefs and lifestyle among community-dwelling middle-aged and elderly individuals in China (*N* = 202).

	**1**	**2**	**3**	**4**	**5**	**6**	**7**	**8**
Perceived susceptibility	1.000	0.265^***^	−0.042	0.181^*^	0.036	−0.101	0.255^***^	−0.157^*^
Perceived severity		1.000	0.117	0.229^**^	0.053	0.066	0.273^***^	−0.094
Perceived benefits			1.000	−0.125	0.196^**^	0.424^***^	0.267^***^	0.415^***^
Perceived barriers				1.000	−0.017	−0.203^**^	0.149^*^	−0.217^**^
General health motivation					1.000	0.291^***^	0.233^**^	0.280^***^
Self-efficacy						1.000	0.203^**^	0.427^***^
Cues to action							1.000	0.074
Lifestyle								1.000

^*^p < 0.05,

^**^p < 0.01,

^***^p < 0.001.

### Measurement model of latent constructs

Henze-Zirkler test showed that the variables used for two CFA were not multivariate normal distribution (positive beliefs: HZ = 25.27, *p* < 0.001; negative beliefs: HZ = 1.28, *p* < 0.001). Therefore, MLM was used for CFA. The measurement model was tested by estimating the association between each item and its hypothetical potential construct (Table 4). For positive beliefs, CFA showed that all the items were significantly loaded with corresponding factors, with first-order standardized factor loads ranging from 0.330 to 0.913 (*p* < 0.001) and second-order standardized factor loads ranging from 0.567 to 0.818 (*p* < 0.001). The goodness of fit of the measurement model was acceptable (CFI = 0.975, TLI = 0.963, RMSEA = 0.034, 90% confidence interval = 0.000–0.067). For negative beliefs, CFA showed that all the items were also significantly loaded with corresponding factors, with first-order standardized factor loadings ranging from 0.490 to 0.944 (*p* < 0.001) and second-order standardized factor loadings ranging from 0.428 to 0.641 (*p* < 0.001). The goodness of fit of the measurement model was acceptable (CFI = 0.988, TLI = 0.985, RMSEA = 0.030, and 90% confidence interval = 0.000–0.054). The detailed data are shown in Table 4.

**Table 4:** Unstandardized and standardized loading for second-order measurement model of positive beliefs and negative beliefs (*N* = 202).

**Parameter estimate**	**Unstandardized loading (*SE*)**	**Standardized loading (*SE*)**
Positive beliefs second-order confirmatory factor analysis model Fit indices: CFI = 0.975, TLI = 0.963, RMSEA = 0.034, 90% CI: 0.000-0.067		
Self-efficacy → SE 1	1.000	0.913 (0.035)^***^
Self-efficacy → SE 2	0.864 (0.098)^***^	0.894 (0.049)^***^
Perceived benefits → Benefit 1	1.000	0.677 (0.074)^***^
Perceived benefits → Benefit 2	0.985 (0.129)^***^	0.783 (0.050)^***^
Perceived benefits → Benefit 3	0.399 (0.121)^**^	0.543 (0.080)^***^
Perceived benefits → Benefit 4	0.823 (0.167)^***^	0.838 (0.069)^***^
General health motivation → HealthM 1	1.000	0.330 (0.078)^***^
General health motivation → HealthM 2	0.628 (0.269)^*^	0.596 (0.126)^***^
General health motivation → HealthM 3	1.160 (0.173)^***^	0.566 (0.105)^***^
General health motivation → HealthM 4	0.803 (0.204)^***^	0.757 (0.099)^***^
Positive belief → Self-efficacy	1.000	0.818 (0.130)^***^
Positive belief → Perceived benefits	0.515 (0.224)^*^	0.676 (0.152)^***^
Positive belief → General health motivation	0.325 (0.139)^*^	0.567 (0.157)^***^
Negative beliefs second-order confirmatory factor analysis model Fit indices: CFI = 0.988, TLI = 0.985, RMSEA = 0.030, 90% CI: 0.000-0.054		
Perceived barriers → Barrier 1	1.000	0.738 (0.056)^***^
Perceived barriers → Barrier 2	0.979 (0.136)^***^	0.705 (0.061)^***^
Perceived barriers → Barrier 3	0.977 (0.155)^***^	0.690 (0.073)^***^
Perceived barriers → Barrier 4	0.910 (0.148)^***^	0.647 (0.075)^***^
Perceived susceptibility → Sus 1	1.000	0.812 (0.033)^***^
Perceived susceptibility → Sus 2	1.193 (0.069)^***^	0.944 (0.023)^***^
Perceived susceptibility → Sus 3	1.053 (0.070)^***^	0.852 (0.028)^***^
Perceived susceptibility → Sus 4	0.690 (0.076)^***^	0.582 (0.055)^***^
Perceived severity → Sev 1	1.000	0.710 (0.050)^***^
Perceived severity → Sev 2	0.924 (0.109)^***^	0.691 (0.047)^***^
Perceived severity → Sev 3	0.658 (0.089)^***^	0.552 (0.055)^***^
Perceived severity → Sev 4	0.850 (0.102)^***^	0.635 (0.053)^***^
Perceived severity → Sev 5	0.651 (0.105)^***^	0.490 (0.069)^***^
Negative belief → Perceived barriers	1.000	0.428 (0.120)^***^
Negative belief → Perceived susceptibility	1.199 (0.470)^*^	0.521 (0.119)^***^
Negative belief → Perceived severity	1.683 (0.708)^*^	0.641 (0.135)^***^

^*^p < 0.05,

^**^p < 0.01,

^***^p < 0.001.

### Structured path model of health beliefs, lifestyle, and cognitive aging grade

**Figure 2:**
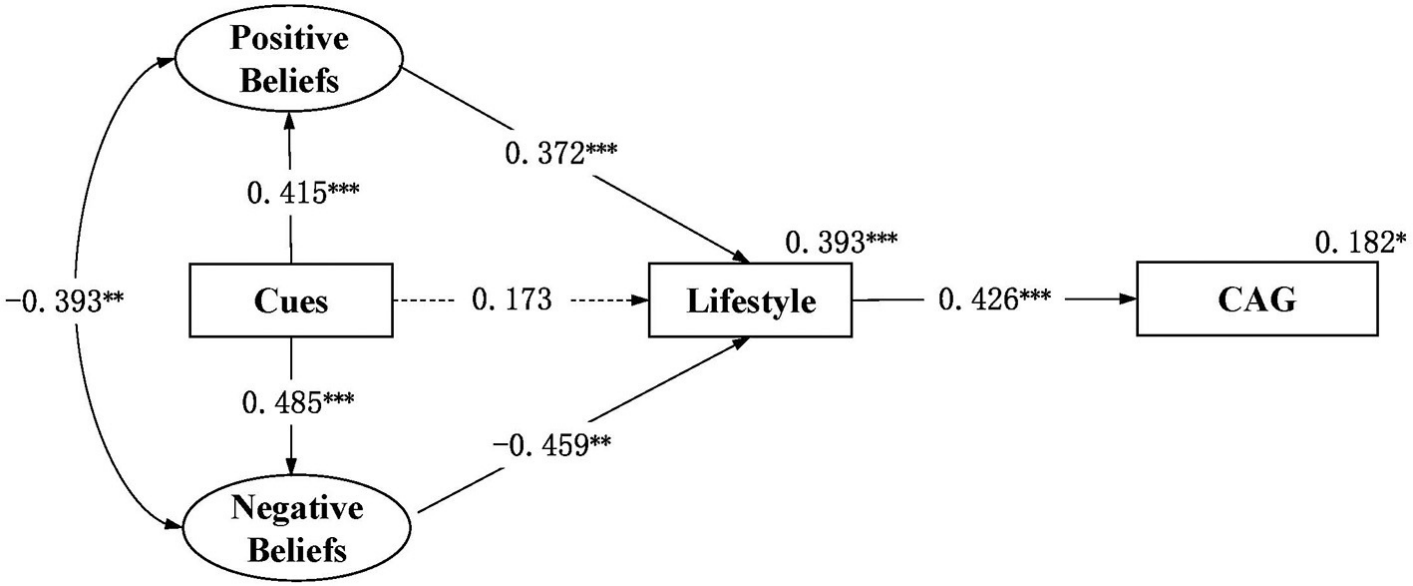
Structured path model of health beliefs, lifestyle and cognitive aging grade among community-dwelling middle-aged and elderly individuals in China (*N* = 202). *p < 0.05, **p < 0.01, ***p < 0.001; Fit index: RMSEA = 0.036, 90% CI 0.023–0.047, CFI = 0.907, TLI = 0.894; CAG, cognitive aging grade; Cues, cues to action. Significant paths were shown in solid lines for simplicity. Dotted lines were the primary paths of interest but not statistically significant. All path coefficients shown were standardized. The oval represents a latent construct measured by multiple items which are not shown in the diagram for simplicity.

Figure 2 shows an acceptable model with CFI = 0.907, TLI = 0.894, RMSEA = 0.036, and 90% confidence interval (CI) = 0.023–0.047. Positive beliefs significantly correlated with the negative beliefs (*R* = –0.393, *p* = 0.002). Statistical significance was shown in the path from cues to action to positive beliefs (β = 0.415, *p* < 0.001) and negative beliefs (β = 0.485, *p* < 0.001), the path from positive beliefs (β = 0.372, *p* < 0.001) and negative beliefs (β = –0.459, *p* = 0.003) to lifestyle, and the path from lifestyle to cognitive aging grades (β = 0.426, *p* < 0.001), supporting hypothesis 1 and 2. There was no statistically significant difference in the path from cues to action to lifestyle (β = 0.173, *p* = 0.223), which was inconsistent with hypothesis 2 that “cues to action can directly affect lifestyle”. The model explained 39.3 and 18.2% of the differences in lifestyle and cognitive aging grades, respectively.

Table 5 shows the indirect effects of health beliefs on lifestyle and cognitive aging grades. Positive beliefs had indirect positive effects on cognitive aging grades (β = 0.158, *p* = 0.005). Negative beliefs had indirect negative effects on cognitive aging grades (β = –0.196, *p* = 0.014). Cues to action had indirect positive effects on lifestyle (β = 0.154, *p* = 0.002) and cognitive aging grades (β = 0.066, *p* = 0.015), as well as had indirect negative effects on lifestyle (β = −0.223, *p* = 0.019) and the cognitive aging grades (β = –0.095, *p* = 0.033), supporting hypothesis 3. However, the total influence of cues to action on lifestyle and cognitive aging grades is not statistically significant.

**Table 5:** Indirect and total effects of positive belief, negative belief and cues to action on lifestyle and cognitive aging grade among community-dwelling middle-aged and elderly individuals in China (*N* =202).

**Total pathway**	**Total indirect effect**		**Specific pathway**	**Indirect effect of path**		**Total effect**	
	**β**	***P***		**β**	***P***	**β**	***P***
Positive belief → CAG	0.158	0.005	Positive belief → Lifestyle → CAG	0.158	0.005	0.158	0.005
Negative belief → CAG	−0.196	0.014	Negative belief → Lifestyle → CAG	−0.196	0.014	−0.196	0.014
Cues → Lifestyle	−0.068	0.603	Cues → Positive belief → Lifestyle	0.154	0.002	0.104	0.158
			Cues → Negative belief → Lifestyle	−0.223	0.019		
Cues → CAG	0.044	0.179	Cues → Lifestyle → CAG	0.074	0.232	0.044	0.179
			Cues → Positive belief → Lifestyle → CAG	0.066	0.015		
			Cues → Negative belief → Lifestyle → CAG	−0.095	0.033		

CAG, cognitive aging grade; Cues, cues to action.

## Discussion

In this study, the participants were divided into three groups: successful cognitive aging, usual cognitive aging, and unsuccessful aging, based on the study of Hartley et al. (6). According to the HBM, this study explored the relationship among beliefs, lifestyle, and cognitive aging grades.

In this study, 8.4% of the participants achieved successful cognitive aging, which was lower than that (16.0%) in the study of Hartley et al. (6). This difference could be related to regional, population, economic, and cultural differences. Currently, no comprehensive and statistical data exist on the proportion of successful cognitive aging in previous studies; however, some studies have demonstrated the proportion of successful memory aging to be approximately 6–40% (29). Univariate analysis showed that the lower the educational level and monthly incomes, the lower the cognitive aging grade. Education status and income are both socioeconomic status (SES) factors. A previous study suggested that low SES is a risk factor for cognitive impairment in older adults (30). Low SES may exacerbate unequal cognitive impairment among older adults, because people with higher SES often have more opportunities to diagnose cognitive impairment, rectify adverse factors, and avoid further deterioration in cognitive function than those with lower SES (31). Such differences in access to medical resources may accumulate over time (32).

The results of this study suggest that a cognitive-related healthy lifestyle has a direct and positive impact on cognitive aging grades. The cognitive-related healthy lifestyle explained 18.2% of the variation in cognitive aging in this study. Notably, dementia is mainly controlled by age and genetic factors (33). Only 40% of cases of dementia are attributable to a combination of modifiable risk factors, including not only lifestyle factors but also traumatic brain injury, air pollution, and other chronic diseases (8). Therefore, the explanation of the rate of variation in this study (18.2%) is reasonable and understandable. Although the impact of lifestyle interventions on individuals may be modest, modifying the individual's lifestyle could have a greater social impact owing to its superior cost-effectiveness compared with other modifiable factors. An increasing number of studies (9, 34–36) have focused on lifestyle interventions for cognitive improvement, and modifying the lifestyle concerning physical activity, smoking, alcohol consumption, diet, social activities, and the management of chronic diseases, such as hypertension, diabetes, obesity, and dyslipidemia, is widely recommended. The LDRR used in this study was also developed according to the recommendations of related studies and guidelines and was more targeted than the general lifestyle scale.

In this study, the health beliefs were divided into three dimensions, including positive beliefs, negative beliefs, and cues to action, based on the study by Li Hua et al. (18), and the HBM. The path analysis in this study showed the specific path and mechanism of these beliefs on lifestyle and cognitive aging grades, as well as the direct and indirect influences among them. Health beliefs accounted for 39.3% of the variation in lifestyle, suggesting that interventions with health beliefs may effectively promote the adoption of cognitive-related healthy lifestyles. In this study, positive beliefs included perceived benefits, general health motivation, and self-efficacy, which described people's concerns about their own health, recognition of the benefits of changing lifestyles, and confidence in adhering to the lifestyle (13). We found that these positive beliefs have a direct and positive impact on lifestyle and indirectly contribute to successful cognitive aging.

This study also showed that negative beliefs had a direct and negative impact on lifestyle and further indirectly impeded the achievement of successful cognitive aging, which was different from the usual cognition of the HBM. According to the HBM (19), perceived susceptibility and perceived severity are used to describe people's perception of threats to diseases, which could stimulate the adoption of a healthier lifestyle to reduce threats. However, considering cognitive aging and dementia prevention, perceived susceptibility and perceived severity may have a hindering effect rather than a promoting effect on people's adoption of healthy behaviors. This could be explained by the concept of dementia worry (DW). DW is defined as an emotional response to the perceived threat of dementia (37). A moderate level of DW leads to adaptive responses, whereas a high level of DW leads to maladaptive responses. Thus, people who perceive acceptable fear and risk of dementia may be more willing to adopt a cognitive-related healthy lifestyle; in contrast, people who are too fearful concerning dementia and even engage in dementia risk reduction may be more willing to avoid behavioral change (17). In addition, previous studies have found that negative perceptions of dementia and aging are associated with DW, including dementia stigma and negative age-related stereotypes (38–40), which may further influence cognitive-related lifestyle changes. The results of this study suggest that the negative effects of negative beliefs on health behaviors should be considered in educational programs for dementia prevention and cognitive aging, especially for people at a high risk of dementia (e.g., those with a family history of dementia). When people present obvious perceived threat (susceptibility and severity) and concern for dementia, medical focus should be transferred to provide feasible interventions on the perceived benefits and self-efficacy to enhance the belief that dementia can be prevented and controlled.

This study found that both positive and negative beliefs were promoted by cues to action. Cues to action are factors that remind individuals to participate in healthy behaviors. Few studies have explored the role of cues to action. Some studies have found that cues to action may indirectly affect behaviors through other health beliefs or may directly affect behaviors (41). Cues to action reportedly work mainly through perceived threats (19). In the MCLHB-DRR, cues to action include amnesia symptoms, dementia risk, media publicity of dementia-related knowledge, and family history of dementia. In this study, cues to action may promote negative beliefs through amnesia symptoms, dementia risk, and a family history of dementia, which may trigger people's perception of dementia threat (perceived susceptibility and severity). Furthermore, cues to action may promote positive beliefs through media publicity, which could provide information on how to adhere to healthy lifestyles and reduce the risk of dementia. However, the overall effect of cues on lifestyle could be weakened because positive and negative beliefs have opposing effects on lifestyle. These results further suggest that, in lifestyle intervention programs, education and advocacy should be used as incentives to reduce negative beliefs and promote positive beliefs.

This study provided evidence for the development of public health interventions. First, community cognitive health management should pay more attention to older adults with limited SES. It would be better to develop targeted healthy lifestyle interventions for older adults with limited SES based on their specific conditions, available resources, and barriers to maximize their use of public medical resources. Moreover, reducing the negative impact of negative beliefs on healthy lifestyles should be recommended in media publicity and health education programs. Targeted interventions on general health motivation, perceived benefits, and self-efficacy may be better for people with high perceived susceptibility and severity (e.g., people with a family history of dementia). It may be necessary to employ these targeted interventions to improve people's awareness of the preventability and controllability of dementia and promote positive belief for adhering to a healthy lifestyle. Finally, interventions can be designed from a more positive perspective to promote successful cognitive aging and encourage people to adopt a more positive attitude toward a cognitive-related healthy lifestyle.

This study had certain limitations. First, owing to the lack of the earlier cognitive function data of the participants, this study only explored the cognitive aging grades from a cross-sectional perspective and classified the cognitive aging grades based on the general cognitive function of community residents who were middle aged and older. With regard to measurement tools, MoCA, as a cognitive screening scale, is inadequate in measuring the general cognitive function of subjects. Moreover, the classification of cognitive aging grade was based on standard score of MoCA, which is the relative standard distance between an individual's cognitive score and the average cognitive score of the group. The cutoff value of cognitive aging grade divided by this method was not constant, and it will vary with samples of different characteristics, which is not conducive to comparison with other studies. Additionally, this classification could hardly explore the rate and trajectory of cognitive decline, and could be biased for different individuals. Second, a single-center survey and limited sample size were employed in this study owing to the limitation of human and economic resources, as well as the barriers brought about by the coronavirus disease 2019. Lastly, considering accessibility, only community residents who had attended free physical examinations in the community health service center were recruited in this study. Compared with those who had not attended free physical examination in the community health service center, participants in this study may have possessed higher levels of health literacy and higher acceptance of cognitive-related healthy lifestyles, which could have resulted in some overestimation in this study for the lifestyle among community residents who were middle aged and older. In the future, the follow-up studies are warranted to further explore successful cognitive aging in individuals from a longitudinal perspective. And additional studies with a larger sample size and wider sampling area, and more fine-grained neuropsychological assessment methods (e.g., attention, speed of information processing, executive function, and episodic memory) should be conducted to further test the model.

## Conclusions

In this study, a limited number of participants achieved successful cognitive aging based on the criteria of this study. Moreover, lifestyle had a direct and positive impact on the cognitive aging grades. Positive beliefs had a positive effect on lifestyle, thus promoting successful cognitive aging. Negative beliefs had a negative effect and hindered the realization of successful cognitive aging. The results suggested that primary medical staff could strengthen the positive beliefs of community residents and weaken their negative beliefs to guide them to adhere to cognitive-related healthy lifestyles and promote successful cognitive aging.

## Data availability statement

The raw data supporting the conclusions of this article will be made available by the authors, without undue reservation.

## Ethics statement

The studies involving human participants were reviewed and approved by the Ethics Committee of the Army Medical University. The patients/participants provided their written informed consent to participate in this study.

## Author contributions

YY developed the ideas, administrated the project, provided significant academic guidance on manuscript, and revised the manuscript critically for intellectual content. JZ and XL collected the data, conducted the data analysis, interpreted the results, drafted, and revised the manuscript. DG and YP collected the data, edited, and revised the manuscript. HL collected the data and edited the manuscript. All authors agreed to be accountable for the content of the work, read, and approved the final manuscript.

## Funding

This study was supported by a grant from the National Social Science Foundation of China (No. 20BRK039). The funders had no role in the study design, data collection, analysis, and decision to publish or preparation of the manuscript.

## Conflict of interest

The authors declare that the research was conducted in the absence of any commercial or financial relationships that could be construed as a potential conflict of interest.

## Publisher's note

All claims expressed in this article are solely those of the authors and do not necessarily represent those of their affiliated organizations, or those of the publisher, the editors and the reviewers. Any product that may be evaluated in this article, or claim that may be made by its manufacturer, is not guaranteed or endorsed by the publisher.

## Abbreviations

CFA: confirmatory factor analysis
CFI: Comparative fit index
CI: confidence interval
DW: dementia worry
HBM: Health Belief Model
HPLP-II: Health Promoting Lifestyle Profile-II
HZ: Henze-Zirkler test
IQR: interquartile range
LDRR: Lifestyle for Dementia Risk Reduction
MCLHB-DRR: Motivation to Change Lifestyle and Health Behaviors for Dementia Risk Reduction
MoCA: Montreal Cognitive Assessment
ML: maximum likelihood
MLM: maximum likelihood parameter estimates with standard errors and a mean-adjusted chi-square test statistic
RMSEA: root mean square error of approximation
SD: standard deviation
SES: socioeconomic status
TLI: Tucker–Lewis index
WLSMV: weighted least squares with mean and variance adjusted.
